# Prevalence and determinants of ideal cardiovascular health by sex and region—a population-based study in Peru

**DOI:** 10.3389/fcvm.2024.1392579

**Published:** 2025-01-13

**Authors:** Víctor Juan Vera-Ponce, Fiorella E. Zuzunaga-Montoya, Luisa Erika Milagros Vásquez-Romero, Joan A. Loayza-Castro, Eder Jesús Orihuela Manrique, Mario J. Valladares-Garrido, Carmen Inés Gutierrez De Carrillo

**Affiliations:** ^1^ Instituto de Investigación de Enfermedades Tropicales, Universidad Nacional Toribio Rodríguez de Mendoza de Amazonas (UNTRM), Amazonas, Peru; ^2^ Facultad de Medicina (FAMED), Universidad Nacional Toribio Rodríguez de Mendoza de Amazonas (UNTRM), Amazonas, Peru; ^3^ Universidad Tecnológica del Perú, Lima, Peru; ^4^ Universidad Continental, Lima, Peru; ^5^ Oficina de Epidemiología, Hospital Regional Lambayeque, Chiclayo, Peru; ^6^ Oficina de Inteligencia Sanitaria, Red Prestacional EsSalud Lambayeque, Chiclayo, Peru

**Keywords:** heart disease risk factors, epidemiologic factors, non-communicable diseases, public health, ideal cardiovascular health (ICH)

## Abstract

**Introduction:**

Attaining what the American Heart Association terms Ideal Cardiovascular Health (ICVH) is viewed as an essential objective for preventing cardiovascular diseases (CVD).

**Objective:**

To determine the prevalence of ICVH, stratified by sex and region and its associated factors in the adult population of Peru.

**Materials and methods:**

Analytical cross-sectional study. Data were obtained from the Life Stage Food and Nutrition Surveillance Survey (VIANEV). ICVH can be depicted vis-a-vis the seven metrics of the AHA: blood pressure levels, total cholesterol and glucose levels, smoking status, body mass index, physical activity levels, and dietary intake through the consumption of fruits and vegetables. The variable was categorized as deficient/moderate vs. ideal for regression analysis.

**Results:**

Of the 863 participants examined, findings demonstrated that 38.01% had ICVH. The prevalence is trending lower in correlation with rising age and educational attainment levels and for those inhabitants residing at higher elevations. Likewise, statistically significant variations were observable concerning the prevalence of ICVH contingent on the region of residence and marital status, in particular amongst the feminine inhabitants.

**Conclusions:**

These results demonstrate that the frequency of ICVH in the grown-up inhabitants of Peru is comparatively tiny. Things linked to a lesser frequency of ICVH involve increased age, a higher level of education, living at higher altitudes, and staying married to a woman. These discoveries underscore the need to implement prevention and treatment strategies for CVD distinct for each inhabitant team.

## Introduction

Cardiovascular health is a concept of great importance in global public health because of its immediate effect on people's quality of life and its result in worldwide mortality (1). It alludes to the heart and veins' condition and capacity to perform their capacities productively. Maintaining an ideal state of cardiovascular well-being (ICVH) is vital to forestall conditions such as hypertension, coronary illness, and stroke (2).

The amount of ICVH is comparatively tiny in the overall population. In Sweden, a review of the middle-aged populace discovered that only 18.2% of the people attained a perfect condition (3). On the other hand, moderate and marginal states of cardiovascular fitness are widespread in the United States (4). In China, the outcomes were similar (5), while in Mexico, ICVH was found to be low (6). And though specific information regarding the prevalence of cardiovascular health in Peru is limited, one study found that ICVH was very small, attaining only 10.5% (7).

Due to the importance of heart and blood vessel fitness and the variances in how common and tied it is in numerous locations and groups, it is vital to comprehend the present situation of this health part and the things that may be related in Peru. In light of this, the current investigation seeks to 1) understand the prevalence of cardiovascular health in Peru stratified by sex and region and 2) determine the factors associated with this state of cardiovascular health. With these goals, heart and blood vessel fitness in Peru is hoped to be understood. It is also expected to furnish significant data for arranging and executing open health intercessions to improve this nation's heart and blood vessel fitness.

## Methods

### Study design and context

Analytical cross-sectional study. Data were obtained from the Life Stages Food and Nutrition Surveillance Survey (VIANEV) during 2017–2018. The VIANEV survey was implemented and supervised by the National Center for Food and Nutrition (CENAN) of Peru (8). To ensure the quality and transparency of our study, we adhered to the STROBE guidelines (Strengthening the Reporting of Observational Studies in Epidemiology) (9).

### Population, sample, and eligibility criteria

The technical report on VIANEV is found elsewhere (8). In summary, it collected information from three distinct domains: Metropolitan Lima, the capital of Peru, and other urban and rural areas. Data was collected through a stratified, multistage, and probabilistic sampling process, independent in each domain. The sample selection was in two stages. In the first stage, clusters of primary sampling units were randomly selected. In the second stage, households were randomly selected within these clusters, including adults aged 18–59. This sampling process allowed for inferences at the national level in urban, rural, and Metropolitan Lima areas.

Specific exclusion criteria were applied to the present work. Those without the variables that make up the cardiovascular health state were excluded.

### Definition of variables

The American Heart Association (AHA) has established seven metrics to define ICVH, Life's Simple 7. These metrics include blood pressure levels, total cholesterol (TC) glucose, smoking status, body mass index (BMI), physical activity levels, and dietary intake. Each of these metrics is evaluated and classified into one of three categories: ICVH, intermediate cardiovascular health (ICVHint), or poor cardiovascular health (ICVHdef). ICVH is defined as having 5–7 metrics in its optimal state, ICVHint is described as having 3–4 metrics in its optimal state, and ICVHdef is defined as having 0–2 metrics in its optimal state. The variable was categorized as poor/moderate vs. ideal for regression analysis (10).

In this study, ICVH was defined as follows:

1. Blood Pressure: An ideal health state was considered if the individual had no history of high blood pressure and their systolic/diastolic blood pressure was <120/80 mm Hg.
2. Total Cholesterol: An ideal health state if the individual had no history of high cholesterol and their TC was <200 mg/dl.
3. Glucose: An ideal health state was considered if the person had no history of high glucose and their fasting plasma glucose was <100 mg/dl.
4. Smoking: An ideal health state was considered if the individual reported never having smoked or if they were an ex-smoker who quit more than 12 months ago.
5. Body Mass Index: An ideal health state was established if the individual's BMI was <25 kg/m^2^.
6. Physical Activity: According to the IPAQ scale, an ideal health state was considered if the individual engaged in moderate or vigorous physical activity.
7. Diet: An ideal health state was considered if the individual consumed fruits and vegetables ≥5 times/day as a substitute for the perfect diet in the ICH definition, as has been used in previous research (7, 11).

Several factors were explored in this work as potential correlates of ICVH, including an individual's biological sex, age groups ranging from 18 to 29 up to 50 to 59 years old, relationship status of single or partnered, highest level of education completed of primary through secondary to high school, natural region of residence of Metropolitan Lima, elsewhere along the coast, the Mountain Range, or jungle, whether residence was located in an urban or rural area, socioeconomic classification of poor or not poor, and altitude of place of residence ranging from 0 to 1,499 meters up to 1,500 meters or higher.

According to existing literature, each of these covariables was selected because of its potential to influence cardiovascular health. The VIANEV report (8) reviews how each variable was measured.

The fasting status was verified through participant self-report during the survey interview, following VIANEV protocol guidelines. While glycated hemoglobin (HbA1c) would provide a more comprehensive assessment of glycemic control over time, this parameter was unavailable in the VIANEV survey. Similarly, the original survey did not collect data on secondhand smoke exposure or environmental smoke from cooking and heating sources.

### Statistical analysis

Data analysis was performed using R software, version 4.0.5. Absolute and relative frequencies were used to describe the data. A bivariate analysis was conducted to evaluate the factors associated with cardiovascular health status, separated by sex; the Chi-square test was used. Adjusted prevalence ratios (aPR) and their 95% confidence intervals (CI 95%) were calculated. For this, generalized linear models were used, assuming a Poisson distribution and using logarithmic link functions. A bar graph was also created to visualize the distribution of each component of ideal ICVH globally, according to sex and region of residence.

It should be noted that all analyses were executed considering the complexity of the sample design. This means that stratification, clustering, and sample weights were assessed in all statistical calculations.

### Ethical aspect

This work was carried out using the VIANEV survey datasets, which are freely and publicly accessible online (https://www.datosabiertos.gob.pe/dataset/estado-nutricional-en-adultos-de-18-59-a%C3%B1os-per%C3%BA-2017-%E2%80%93-2018). All personal identifiers were removed from these datasets before publication, thus ensuring the privacy and anonymity of the study participants.

Since this study analyzes existing and completely anonymous data, it was not considered necessary to submit it to an ethics committee review. This stance aligns with ethical guidelines for health research, which state that analyses of existing public datasets that do not contain identifiable information do not require honest review. Nevertheless, all necessary measures were taken to ensure that the analysis was conducted ethically and that the rights and dignity of the original study participants were respected.

## Results

Our work included 863 participants, with a balanced distribution by age group and a slight predominance of women (56.55%). Most lived with a partner (64.54%) and resided in Metropolitan Lima (46.12%). Regarding the educational level, there was an almost equal distribution between those with secondary education (37.95%) and those with a higher education level (38.77%). Regarding alcohol consumption, participants were almost equally divided. Regarding cardiovascular health, only 11.01% of participants had poor cardiovascular health, while 38.01% had an ideal one (Table 1).

**Table 1:** General characteristics of the study participants and prevalence of ICVH.

Characteristic	*N* = 863
Sex	
Female	488.00 (56.55%)
Male	375.00 (43.45%)
Age group	
18–29 years old	239.00 (27.69%)
30–39 years old	216.00 (25.03%)
40–49 years old	214.00 (24.80%)
50–59 years old	194.00 (22.48%)
Civil status	
Single	306.00 (35.46%)
With couple	557.00 (64.54%)
Educational Level	
Until primary	200.00 (23.28%)
Secondary	326.00 (37.95%)
Higher	333.00 (38.77%)
Natural region	
Metropolitan Lima	398.00 (46.12%)
Resy of coast	183.00 (21.21%)
Mountain Range	156.00 (18.08%)
Jungle	126.00 (14.60%)
Alcohol consumption	
No	448.00 (51.91%)
Yes	415.00 (48.09%)
Area of residence	
Rural	285.00 (33.02%)
Urban	578.00 (66.98%)
Wealth index	
No poor	699.00 (81.00%)
Poor	164.00 (19.00%)
Altitude	
0 a 1,499	669.00 (77.52%)
1,500 a más	194.00 (22.48%)
Cardiovascular health	
Poor	95.00 (11.01%)
Intermediate	440.00 (50.98%)
Ideal	328.00 (38.01%)

*n* (%).

Source: self-made.

**Figure 1:**
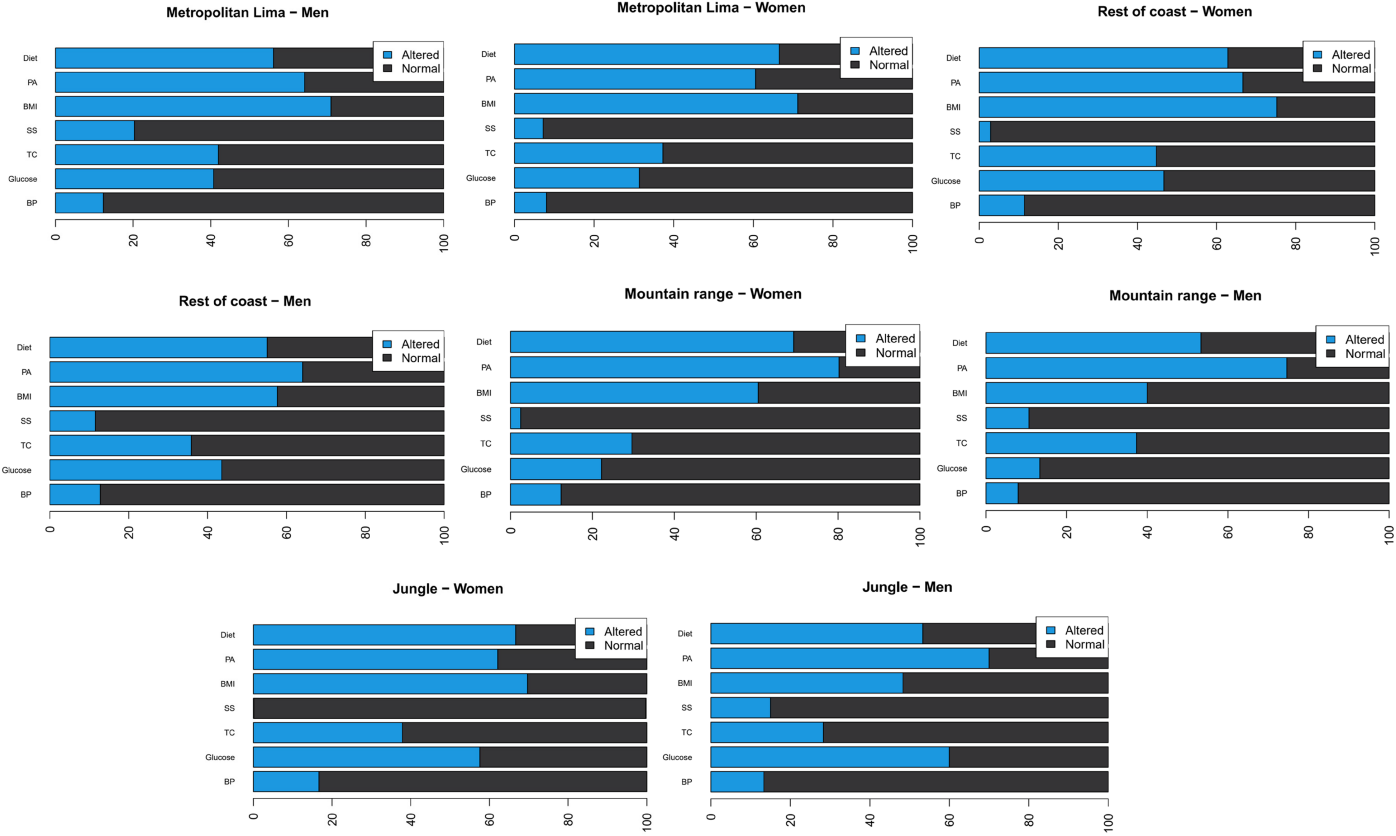
Prevalence (%) of poor/intermediate (light blue) and ideal (dark blue) cardiovascular health metrics by sex and region. BP, blood pressure; TC, total cholesterol; GLU, glucose; SMK, smoking status; BMI, body mass index; PA, physical activity; DIET, dietary intake (fruit and vegetable consumption).

In general, markers of cardiovascular diseases (CVD) show similar patterns across all regions in both males and females. In men, the presence of hypertension is lowest in the Highlands (8%), followed by the Jungle (13.33%), the Rest of the Coast (12.82%), and Metropolitan Lima (12.35%). High glucose is especially prevalent in the Highlands (86.67%) and is significantly lower in the Jungle (40%). Lack of physical activity is more common in the Highlands (74.67%) and the Rest of the Coast (64.1%), while tobacco consumption is higher in Metropolitan Lima (79.63%). In women, the presence of hypertension is lowest in the Jungle (16.67%), closely followed by the Rest of the Coast (11.43%) and Metropolitan Lima (8.05%). High glucose is more common in the Highlands (77.78%) and the Rest of the Coast (53.33%). Lack of physical activity is more common in the Highlands (80.25%) and the Rest of the Coast (66.67%). Tobacco consumption is almost non-existent in the Jungle (0%), while it is higher in Metropolitan Lima (92.8%)—Figure 1.

There was no statistically significant difference between the overall prevalence levels of ICVH among males and females (*p* = 0.295). Table 2 shows the factors associated with ICVH by sex.

**Table 2:** Factors associated with ICVH by sex.

Characteristic	Women			*p*-value	Men			*p*-value
	Ideal *n* = 178	Intermediate *n* = 260	Poor *n* = 50		Ideal *n* = 150	Intermediate *n* = 180	Poor *n* = 45	
Age group				**<0**.**001**				**<0**.**001**
18–29 years old	69.00 (53.91%)	56.00 (43.75%)	3.00 (2.34%)		67.00 (60.36%)	43.00 (38.74%)	1.00 (0.90%)	
30–39 years old	52.00 (41.94%)	63.00 (50.81%)	9.00 (7.26%)		33.00 (35.87%)	50.00 (54.35%)	9.00 (9.78%)	
40–49 years old	31.00 (23.66%)	83.00 (63.36%)	17.00 (12.98%)		30.00 (36.14%)	38.00 (45.78%)	15.00 (18.07%)	
50–59 years old	26.00 (24.76%)	58.00 (55.24%)	21.00 (20.00%)		20.00 (22.47%)	49.00 (55.06%)	20.00 (22.47%)	
Civil status				**<0**.**001**				**<0**.**001**
Single	81.00 (47.65%)	71.00 (41.76%)	18.00 (10.59%)		71.00 (52.21%)	58.00 (42.65%)	7.00 (5.15%)	
With couple	97.00 (30.50%)	189.00 (59.43%)	32.00 (10.06%)		79.00 (33.05%)	122.00 (51.05%)	38.00 (15.90%)	
Educational Level				0.242				0.124
Until primary	41.00 (30.15%)	82.00 (60.29%)	13.00 (9.56%)		33.00 (51.56%)	28.00 (43.75%)	3.00 (4.69%)	
Secondary	58.00 (35.37%)	88.00 (53.66%)	18.00 (10.98%)		63.00 (38.89%)	75.00 (46.30%)	24.00 (14.81%)	
Higher	78.00 (42.16%)	90.00 (48.65%)	17.00 (9.19%)		54.00 (36.49%)	76.00 (51.35%)	18.00 (12.16%)	
Natural region				0.221				**0**.**003**
Metropolitan Lima	95.00 (40.25%)	120.00 (50.85%)	21.00 (8.90%)		52.00 (32.10%)	84.00 (51.85%)	26.00 (16.05%)	
Resy of coast	29.00 (27.62%)	63.00 (60.00%)	13.00 (12.38%)		29.00 (37.18%)	42.00 (53.85%)	7.00 (8.97%)	
Mountain Range	33.00 (40.74%)	42.00 (51.85%)	6.00 (7.41%)		45.00 (60.00%)	23.00 (30.67%)	7.00 (9.33%)	
Jungle	21.00 (31.82%)	35.00 (53.03%)	10.00 (15.15%)		24.00 (40.00%)	31.00 (51.67%)	5.00 (8.33%)	
Alcohol consumption				**0**.**05**				0.436
No	119.00 (39.40%)	159.00 (52.65%)	24.00 (7.95%)		63.00 (43.15%)	64.00 (43.84%)	19.00 (13.01%)	
Yes	59.00 (31.72%)	101.00 (54.30%)	26.00 (13.98%)		87.00 (37.99%)	116.00 (50.66%)	26.00 (11.35%)	
Area of residence				0.334				**0**.**03**
Rural	58.00 (38.16%)	83.00 (54.61%)	11.00 (7.24%)		65.00 (48.87%)	53.00 (39.85%)	15.00 (11.28%)	
Urban	120.00 (35.71%)	177.00 (52.68%)	39.00 (11.61%)		85.00 (35.12%)	127.00 (52.48%)	30.00 (12.40%)	
Wealth index				0.501				0.261
No poor	140.00 (35.99%)	206.00 (52.96%)	43.00 (11.05%)		121.00 (39.03%)	148.00 (47.74%)	41.00 (13.23%)	
Poor	38.00 (38.38%)	54.00 (54.55%)	7.00 (7.07%)		29.00 (44.62%)	32.00 (49.23%)	4.00 (6.15%)	
Altitude				0.147				**<0**.**001**
0 a 1,499	134.00 (34.36%)	214.00 (54.87%)	42.00 (10.77%)		95.00 (34.05%)	149.00 (53.41%)	35.00 (12.54%)	
1,500 a más	44.00 (44.90%)	46.00 (46.94%)	8.00 (8.16%)		55.00 (57.29%)	31.00 (32.29%)	10.00 (10.42%)	

*n* (%).

Pearson's Chi-squared test.

Bold values indicate statistically significant value (<0.05).

Source: self-made.

Table 3 presents the multivariable regression analysis, in which significant differences were observed in the prevalence of cardiovascular risk factors between men and women, as well as among different age groups, marital status, educational level, natural region, area of residence, wealth index, alcohol consumption, and altitude.

**Table 3:** Multivariable regression analysis of the factors associated with ICVH.

Characteristic	Women		*p*-value	Men		*p*-value
	aRP*	95% CI		aRP*	95% CI	
Age group						
18–29 years old	—	—		—	—	
30–39 years old	0.95	0.63, 1.43	0.763	0.6	0.37, 0.96	**0**.**005**
40–49 years old	0.5	0.31, 0.79	**<0**.**001**	0.59	0.34, 0.99	**0**.**006**
50–59 years old	0.52	0.31, 0.84	**<0**.**001**	0.34	0.19, 0.61	**<0**.**001**
Civil status						
Single	—	—		—	—	
With couple	0.76	0.54, 1.08	**0**.**044**	0.79	0.52, 1.21	0.129
Educational Level						
Until primary	—	—		—	—	
Secondary	1.04	0.67, 1.63	0.805	0.68	0.42, 1.10	**0**.**024**
Higher	1.19	0.75, 1.91	0.321	0.59	0.35, 1.02	**0**.**006**
Natural region						
Metropolitan Lima	—	—		—	—	
Rest of coast	0.57	0.35, 0.91	**0**.**005**	0.98	0.58, 1.60	0.904
Mountain Range	0.53	0.26, 1.08	**0**.**027**	1.36	0.67, 2.77	0.239
Jungle	0.62	0.34, 1.09	**0**.**032**	1.04	0.57, 1.85	0.84
Area of residence						
Rural	—	—		—	—	
Urban	0.84	0.50, 1.40	0.391	0.91	0.55, 1.51	0.612
Wealth index						
No poor	—	—		—	—	
Poor	0.95	0.61, 1.46	0.75	0.96	0.59, 1.53	0.799
Alcohol consumption						
No	—	—		—	—	
Yes	0.77	0.55, 1.08	0.051	0.95	0.68, 1.35	0.703
Altitude						
0 a 1,499	—	—		—	—	
1,500 a más	1.82	1.01, 3.24	**0**.**013**	1.29	0.74, 2.25	0.21

aPR, Ajusted prevalence ratio; 95% CI, 95% confidence interval.

Bold values indicate statistically significant value (<0.05).

Source: self-made.

* Adjusted for group age, civil status, educational level, natural region, alcohol consumption, área of residence, wealth index, and altitude.

In women, the prevalence of cardiovascular risk factors was found to decrease significantly with age, being lower in the 40–49-year-old group (aPR: 0.5; 95% CI: 0.31, 0.79) and 50–59-year-old group (aPR: 0.52; 95% CI: 0.31, 0.84) compared to the 18–29-year-old group. Women living with a partner also showed a lower prevalence than single women (aPR: 0.76; 95% CI: 0.54, 1.08). Those living on the coast (aPR: 0.57; 95% CI: 0.35, 0.91), in the Mountain Range (aPR: 0.53; 95% CI: 0.26, 1.08), and in the jungle (aPR: 0.62; 95% CI: 0.34, 1.09) demonstrated a lower prevalence compared to those living in Metropolitan Lima. Those residing at altitudes of 1,500 meters or more showed a higher prevalence (aPR: 1.82; 95% CI: 1.01, 3.24).

The prevalence of cardiovascular risk factors in men also decreased with age, being lower in the 30–39-year-old group (aPR: 0.6; 95% CI: 0.37, 0.96), 40–49-year-old group (aPR: 0.59; 95% CI: 0.34, 0.99), and 50–59-year-old group (aPR: 0.34; 95% CI: 0.19, 0.61) compared to the 18–29-year-old group. Men with a secondary education level (aPR: 0.68; 95% CI: 0.42, 1.10) and higher education level (aPR: 0.59; 95% CI: 0.35, 1.02) showed a lower prevalence vs. those with up to primary education.

## Discussion

### Prevalence of cardiovascular health Status

The prevalence of intermediate ICVH was presented in almost half of those evaluated, while approximately one-tenth had a poor level. The overall levels between men and women were not statistically significant. These results are consistent, on the one hand, and contrast with other recent studies that have examined the prevalence of ICVH in different populations.

For example, a study conducted in the general middle-aged population in Sweden found that only 18.2% of the population reached the ideal state of ICVH. In comparison, 51.9% were classified as intermediate status, a level very similar to ours (3). Meanwhile, Gupta et al. (12) found that under half of their sample (43.1%) had ideal ICVH. Conversely, research conducted in China found shallow values of ideal ICVH (15.7% for men and 6.8% for women). In the study by Ghimire et al. (13), conducted in Nepal, there was a difference of almost ten percentage points in ideal ICVH between both sexes.

Specifically, if we focus on studies in Latin America, the research by Machado et al. (14) and Avecedo et al. found that only 7.8% and 14.3% had ideal ICVH. In Peru, Benziger et al. (7) found that only 10.5% of the evaluated Peruvians had ideal ICVH, unlike our research, in which it was three times more. The discrepancies of the latter may lie in the work of Bezinger et al. (7), who evaluated the population of four cities in the country. In contrast, in the present work, a national database was used.

The differences in ICVH prevalence between different populations may be due to various factors, according to an article by Peters et al. (15). It has been recommended that the patterns over time in cardiovascular danger elements may change between genders, which could account for a few of the distinctions that have been noticed. The researchers' findings revealed that whereas females saw body mass index increase more noticeably relative to males throughout the same timeframe, total cholesterol experienced a diminution more markedly for males in contrast to females. Similarly, Lu and colleagues examined this topic in another investigation. Found that although hypertension is more common in the United States, blood pressure levels are higher in China, which changes ICVH values (16).

### Factors associated with ICVH

Investigations into the connection between advancing age and cardiovascular wellness have revealed that with time, an individual's cardiac and vascular fitness will inevitably fade without preventative measures. The Framingham study discovered that the frequency of cardiovascular danger elements like hypertension, diabetes, obesity, and dyslipidemia amplified substantially right after a person's half-century birthday (17). The findings revealed that as one age, researchers uncovered links between amplified arterial stiffness, thickened inner linings of the carotid arteries, and exacerbated systolic blood pressure (18). The male-female ratio for coronary disease also reverses with age, being higher in young males but later higher in elderly females (19). These studies underline that advanced age, particularly after 65, is significantly associated with an increase in the prevalence of various types of ICVH. This effect seems more pronounced in young men but balances in advanced ages. In turn, it is vital to highlight that, in our work, the decrease in the prevalence of ideal ICVH with age was more pronounced in men than in women. Based on these findings, it is tentatively proposed that the cardiovascular system in males may prove more vulnerable to the deteriorative impacts of advancing years than in females. While further exploration could help validate this proposed explanation, more evidence is required before the hypothesis can be considered corroborated. A 2018 investigation suggested that the dissimilarities between male and female endothelial performances could be pivotal for vascular well-being and the all-encompassing peril of cardiovascular illnesses throughout one's lifetime (20).

Recent studies have explored the potential variances between sexes in terms of how marital status may impact achieving an optimum level of cardiovascular well-being. The work of Manfredini et al. (21), a large-scale investigation of over 21,000 individuals across the United States, revealed that married women or those in long-term partnerships were more inclined to attain ideal cardiovascular risk factors, including appropriate blood pressure levels when juxtaposed against their unmarried, divorced, or widowed associates. A further investigation conducted by the research team of Wang and colleagues was also published (22) found that participants with a higher score of ideal cardiovascular health metrics had a lower risk of having suboptimal health status. The findings from these investigations indicate that relationship status may considerably influence cardiovascular well-being, especially among females (23). While further exploration could provide deeper insight into how this association works and the extent to which additional determinants, like lifestyle choices or consistency of care, may also impact it, the whole nature of such a link remains uncertain.

An intriguing finding in our study was the decrease in cardiovascular risk factors with age in both men and women, which appears to contrast with traditional cardiovascular epidemiology literature. Several mechanisms might explain this unexpected pattern in the Peruvian context. First, this could reflect a survival effect, where individuals with better cardiovascular health are more likely to survive in older age groups. Second, our cross-sectional design captures different birth cohorts simultaneously—younger generations may have adopted more contemporary lifestyle habits (increased sedentary behavior, processed food consumption, and stress levels) that negatively impact cardiovascular health. Third, older adults in Peru might maintain more traditional lifestyle patterns, including higher consumption of conventional foods and more physical activity in daily life, particularly in non-urban areas. Fourth, there might be increased health awareness and better health-seeking behaviors among older adults with more frequent contact with healthcare services. Additionally, the higher prevalence of risk factors in younger adults could signal a concerning trend that warrants public health attention, as it may predict a higher burden of cardiovascular disease in future decades. These findings highlight the importance of early preventive interventions targeting younger age groups in Peru.

Our manuscript found that the likelihood of having an ideal ICVH decreases as the educational level increases. This finding echoes conclusions previously explored in earlier studies examining this same subject matter. A substantial part of the academic level differences seen in cardiovascular and all-cause mortality between Finnish men and women was discovered by the study from Finland to stem from variations in health behaviors such as engagement in smoking, the consumption of vegetables, and physical activity participation (24). Likewise, research in China observed that the pathways of cardiovascular wellness ratings over time were connected to succeeding chances of cardiovascular illness (25). Similarly, a study in Italy found that the burden of cardiovascular diseases and their risk factors remained high and required continuous appropriate action at the community and individual levels (26). Finally, a study in Turkey found a possible association between an adverse lipid profile, specific dietary patterns, and a higher level of parental education among school children (27). These results underscore the importance of education on maintaining cardiovascular health.

It was found that the natural region in which women live has a significant association with their ICVH. Specifically, females living on the coast (apart from the Lima metropolis), in the mountains, or in the jungle are less likely to have ideal ICVH vs. those living in the Lima metropolis. These findings are consistent with literature suggesting that geographical location may impact cardiovascular health. For instance, the systematic work examining the effects of exposure to environmental noise on hypertension, blood pressure, and ischemic heart diseases found that the results and exposure patterns may depend on gender as determined by the various social, economic, and cultural factors within society (28). While the natural region did not correlate with ICVH in the male participants of our research, other considerations remained unclear and warranted further exploration. While this finding indicates other drives may carry more weight in shaping men's internal cranial volumes or the association between geography and brain size proves more intricate for males, the relationship demands further scrutiny to unravel potentially nuanced influences.

Our findings revealed that females inhabiting elevations no less than 1,500 meters demonstrated an 82% higher probability of possessing ICVH than those from zero to 1,499 meters above sea level. Indeed, the finding that altitude may confer cardiovascular benefits corroborates prior work, implying such a protective impact, and represents an exciting development aligning with extant literature on this topic (29). Researchers in Austria discovered residence at moderate elevations appears increasingly protective against mortality from assorted causes, notably cardiovascular diseases. At the same time, a separate expedition to Everest found nearly half the hikers had pre-existing orthopedic or cardiovascular conditions (30). Preventive measures and education about altitude sickness deserve special attention.

### Limitations of the study

Though the findings offer valuable perspective, one must consider various qualifiers when evaluating their full import. First, the study's cross-sectional nature cannot establish causal relationships between associated factors and ideal cardiovascular health status. Consequently, one should view the outcomes as interrelated factors rather than decisive causes, as correlation does not necessarily imply causation. While adjustments have been made for multiple influences in our examinations, the potential for some skewness remains as not all pertinent influences may have been adequately accounted for or quantified entirely due to restricted data. While certain constraints remain, our findings offer meaningful insight into the occurrence and correlates of ideal heart wellness in Peruvians, as characterized by established health indicators.

Additionally, some methodological constraints should be noted. First, fasting glucose measurements relied on self-reported fasting status, which could introduce recall bias. While HbA1c might provide a more reliable assessment of glycemic status, these data were unavailable in the VIANEV survey. Furthermore, the study did not account for secondhand smoke exposure or environmental smoke from cooking and heating sources, which could potentially impact cardiovascular health, particularly in rural areas where traditional cooking methods are common. Future studies in Peru should consider incorporating these important environmental exposures to provide a more comprehensive assessment of cardiovascular risk factors.

## Conclusions

Our work provides a comprehensive view of the prevalence and factors associated with ideal cardiovascular health status in the adult population of Peru. The study found that ICVH is comparatively infrequent, most notably for older individuals, those possessing higher educational attainment levels, and residents dwelling at more considerable elevations.

These results indicate the need to implement prevention and control strategies for cardiovascular diseases targeted at each population group. Cardiovascular health education must specifically target improving healthy lifestyle promotion for older individuals, those with higher educational attainment, and inhabitants of more elevated elevations, as it is particularly crucial to do so.

Furthermore, our findings suggest that public health interventions must consider regional and gender differences in the prevalence of ideal ICVH. For example, those aimed at women could promote cardiovascular health among married women. In contrast, interventions aimed at those living on the coast and in the mountains could focus on addressing these regions' specific cardiovascular risk factors.

Finally, our results highlight the importance of further research to understand better the factors contributing to the prevalence of ideal ICVH in Peru. Through further exploratory research, insights could be gleaned that may serve to more astutely guide the shaping of public health policies and design of improved tactics for both thwarting and containing the spread of cardiovascular diseases across Peru.

## Data Availability

The data supporting the findings of this study can be accessed at the follow link: https://www.datosabiertos.gob.pe/dataset/estado-nutricional-en-adultos-de-18-59-a%C3%B1os-per%C3%BA-2017-%E2%80%93-2018.
